# Conditions for the emergence of circumnutations in plant roots

**DOI:** 10.1371/journal.pone.0252202

**Published:** 2021-05-26

**Authors:** Ilya Loshchilov, Emanuela Del Dottore, Barbara Mazzolai, Dario Floreano

**Affiliations:** 1 Laboratory of Intelligent Systems, École Polytechnique Fédérale de Lausanne, Lausanne, Switzerland; 2 Center for Micro-Biorobotics, Istituto Italiano di Tecnologia, Pontedera, Italy; Vrije Universiteit Amsterdam, UNITED KINGDOM

## Abstract

The plant root system shows remarkably complex behaviors driven by environmental cues and internal dynamics, whose interplay remains largely unknown. A notable example is circumnutation growth movements, which are growth oscillations from side to side of the root apex. Here we describe a model capable of replicating root growth behaviors, which we used to analyze the role of circumnuntations, revealing their emergence I) under gravitropic stress, as a combination of signal propagation and sensitivity to the signal carriers; II) as a result of the interplay between gravitropic and thigmotropic responses; and III) as a behavioral strategy to detect and react to resource gradients. The latter function requires the presence of a hypothetical internal oscillator whose parameters are regulated by the perception of environmental resources.

## Introduction

A crucial question in plant development is how external cues are translated into specific growth patterns and how these are internally coordinated [1]. Plant hormones, such as auxin, are involved in virtually all aspects of plant development [2], including directional growth response (tropisms) [3], the control of plant architecture [4, 5], stress response [6], and embryo development [7]. The differential distribution of the auxin hormone regulates the signaling network and the plant organ’s responses to stimuli [1, 8], such as the inhibition or stimulation of cell elongation, which leads to the bending of the organ. Growth occurs in the apical region of the root, which includes cell division and elongation zones [9]. The transition between these two zones depends on the auxin profile, with the transition border defined by the minimum auxin level [10]. The unequal rate of growth on opposite sides is the mechanism underlying circular or elliptical movements, called circumnutations [11], with the highest rate of differential elongation localized at the middle of the elongation zone [12]. While auxin is expected to play an important role in circumnutation movements, there is a wide range of other chemical regulators (peptides, RNAs, ions, and metabolites [13]) and corresponding signaling processes that affect plant growth and development [14] and can possibly play a role in the emergence of circumnutation movements. Previous hypotheses and experiments described circumnutations as a response to gravity [15], tactile stimulation [12], or internal ion flux oscillations [16], ascribing to these movements a possible role in anchoring [17] and soil penetration [18]. The latter hypothesis was translated into a control strategy of a plant root-inspired robot moving in soil [19, 20]. However, given the complexity of signal pathways in plants [21–23], the design and analysis of biological experiments to disambiguate the nature and the role of circumnutations in plant roots still remain very challenging. Formal and numerical models of plant root growth can help better understand and assess how incoming signals translate into growth movements and generate circumnutations.

Quite extensive work has been carried out to model and analyze the kinematics of plant shoots [24–28] and their circumnutations [29], even considering multiple stimuli and auxin-mediated signaling pathways. However, root and shoot are two naturally distinct organs, with design and functional divergences, deserving dedicated attention. Some attempts have been made to describe plant roots 2-D kinematics under gravity effects [30], considering gravity and water [31] or analyzing cells geometric factors and mechanical properties driving tissue extensibility and root bending [32].

Other current plant root models are based on functional-structural approaches [33–35], which mainly address root branching and arrangement in space, internal fluxes (e.g., water, sugars), or auxin-specific distribution [36, 37]. Nonetheless, none of the root models explains 3-dimensional root bending behaviors while accounting for root zonation in response to a combination of environmental stimuli (e.g., identifying developmental zones along the root where the response is actuated). In contrast, root zonation is essential for coordinated cell differentiation and fast tropic environmental responses [9]. Plant growth is mainly regulated by a dynamic change in the cell activities of transcription factors and protein regulators interconnected to hormone biosynthesis [38]. Such internal processes have been compared to computational circuits analogous to nervous systems [39], and a wide range of biological insights have been leveraged to promote the analogy between a neural network and the plant root seen as a network of cells linked by biochemical connections [40, 41]. However, no formalization and computational predictions have been described so far. Here we propose a model where the maximal root growth rate and position can be adjusted and thus analyzed on different developmental zones of the apex, regulated by a signaling network that transmits excitation and inhibition signals akin to an artificial neuronal network. The comparison between neural networks and plants has created a great debate in the scientific community [42–45], opening new frontiers in the understanding of plants [46]. Despite this, it is not our intention to assert a close analogy between plants and neural networks as in animals, foraging the debate, but rather to analyze some of the plant’s behaviors through their description with artificial networks that can well describe plant’s behavior functions. Specifically, we want to examine the conditions for the emergence of circumnutations in plants during growth, providing a general model whose parameters can then be tuned to ascribe from specific plant species. The model decomposes the complex process of signaling involved in root-environment interactions into simpler components (e.g., responses to gravity, touch, nutritional resources) that can be studied, both individually and in interaction, to discriminate the effect of each component in the emergence of circumnutations. The proposed model can reproduce observable growth patterns of living plant roots and reveal the emergence of circumnutation movements as both a consequence of gravitropic stress and as a possible result of the interplay between gravitropic and thigmotropic stimulation. Ultimately, under the assumption of an internal oscillator dictating the presence of circumnutations, these movements are investigated in this paper for the first time as a possible strategy to detect and react to resource gradients, with resource signals affecting circumnutation period and amplitude. In the following, the model will be first presented and described. Then, the results of different model parameters investigation will be discussed, followed by conclusive remarks.

## Materials and methods

**Fig 1:**
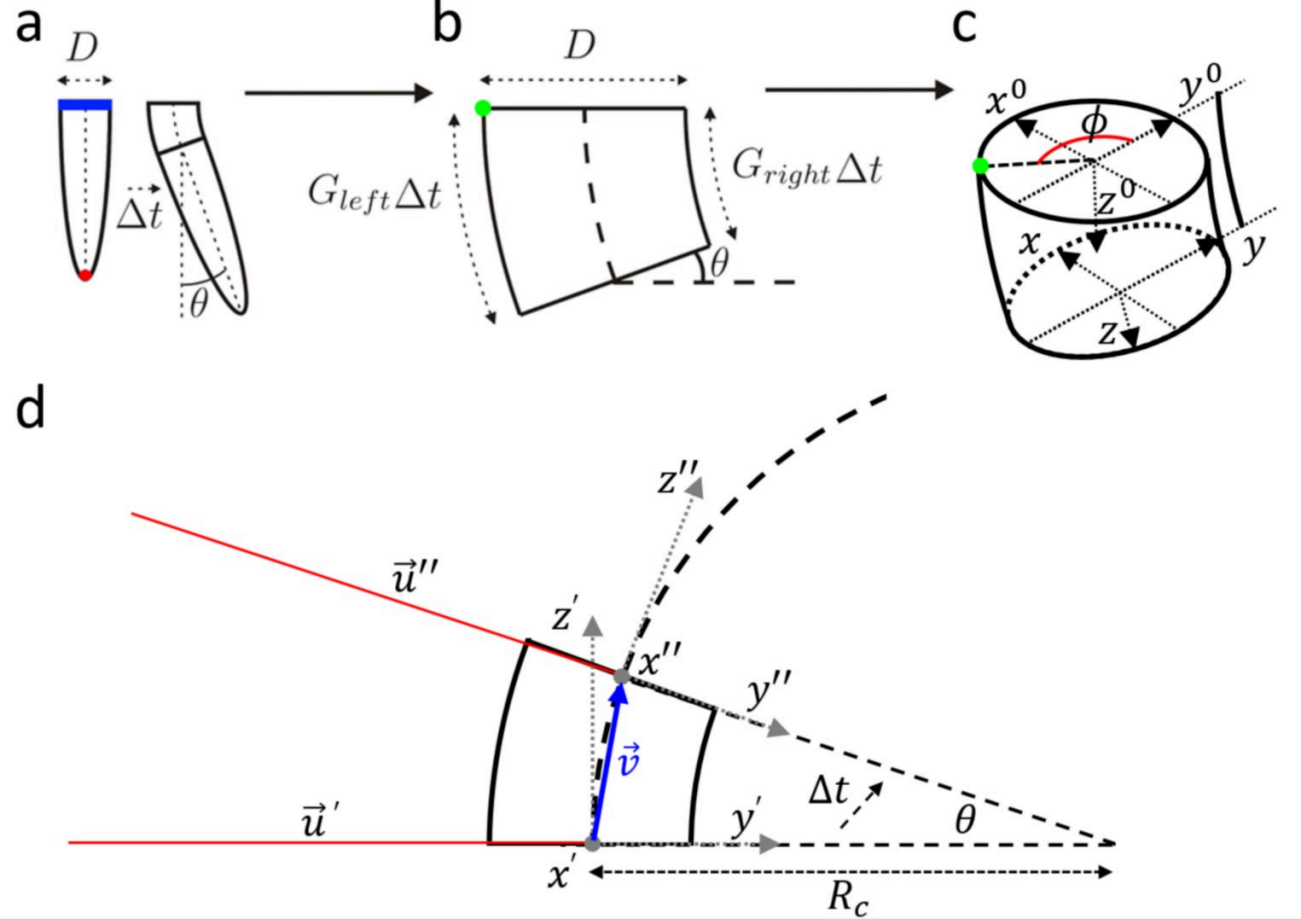
Schematic of root parameters and growth kinematics. The mechanism of differential growth for a single time step (Δ*t*) is summarized for our modification of the 2-dimensional model of Tsutsumi [31]. (a) The root before and after a bend of *θ*. The red dot identifies the tip of the root, and the blue line highlight the diameter *D*. (b) A close view of the region with the bending. The differential growth is achieved by a different growth rate (*G*_*left*_>*G*_*right*_ in this example) obtained at opposite sides of the root. (c) A 3-D view of the cross-section of the root, the local coordinate system is shown before *Ox*^0^*y*^0^*z*^0^ and after *Oxyz* the differential growth. This 3-D section is representative for the shape of segments defined on the root at each iteration. Outside of the root is drawn the reference curve location (black line in the direction of the *y* axis), the point having highest growth rate (green dot) and the corresponding *ϕ* angle, which corresponds to the angle between the *y* axis and the point of highest growth rate. (d) The kinematics for the same growth step.

The proposed 3-D growth model with an embedded signaling network was inspired by Tsutsumi’s model in 2-D [31], in which the growth process is modeled by controlling the elongation rates (*G*_*left*_ and *G*_*right*_ in Fig 1) at two opposite sides of the flank behind the root tip (red dot in Fig 1). Different elongation rates produce differential growth generating a curvature, with a bending angle *θ* after an interval of time Δ*t*, given by:

$$\theta =\frac{\left|G_{left}\Delta t-G_{right}\Delta t\right|}{D},$$ (1)

where *D* is the root diameter (blue line in Fig 1). When *G*_*left*_ equals *G*_*right*_, a straight growth is achieved. The average growth rate is then defined as *G*_*avr*_ = (*G*_*left*_+*G*_*right*_)/2. According to [47], root growth responses change when the roots are under environmental stresses. Thus, the root growth direction can be coordinated by adjusting *G*_*left*_ and *G*_*right*_ over time according to environmental signals. Whereas in a 2-D case, the allowed angles (*ϕ*) along the circumference of the root are only *ϕ* = 0° for the right side and *ϕ* = 180° for the left side, in our 3-D model the growth rates can be defined for any angle *ϕ* in the range [−180°,+180°] around the circumference of the root (Fig 1C).

The proposed 3-D model allowed simulating a plant root’s growth in response to the current state affected by internal/external signals that may originate at various root positions and are transmitted via parametrized signaling pathways. To describe the rate of axial root elongation, we introduced the *stimuli growth response surface G*_*s*_(*t*,*d*,*ϕ*) function (detailed below in the text) defining the rate of axial elongation of cells on the root surface. *G*_*s*_(*t*,*d*,*ϕ*) can vary in time *t*, for a distance *d*∙*D* from the tip along the root centerline, and with angle *ϕ* w.r.t. the *y* axis (Fig 1C). To easily visualize the *y* axis direction, a black line, which we call reference curve, is also plotted in our graphs (black lines in Fig 1C and S1C, S1F and S1I Fig) that follows the root trajectory outside of the root diameter.

The initial state of the root is constructed as a 3-dimensional straight object composed of *n* 3-dimensional sections, having a cylindrical shape (Fig 1C), whose diameters *r*(*d*) are computed as a function of their distance *d* from the tip according to:

$$r(d)=\left\{ \begin{array}{c} 0.5{D(\frac{d}{d_{cap}})}^{p_{cap}},if\;d<d_{cap}\\ 0.5D,otherwise \end{array}\right.,$$ (2)

where *d*_*cap*_ = 1.5 defines the threshold root cap distance and *p*_*cap*_ = 0.4 defines the cap curvature. Considering that in the most studied model plants like *Arabidopsis thaliana* or *Zea mays*, the root diameter is constant over the elongation zone, except for the very apical region [48, 49], and since also we are not studying morphological alterations (i.e., radial expansion), but only motion patterns generated during growth, our assumption on the root diameter can be considered valid.

The length of the initial root is defined by the length of the elongation region (set to 6.0*D* in our simulations). To achieve sufficient simulation precision, the number of sections *n* should be sufficiently large and the simulated time-step Δ*t* should be sufficiently small. We limit our observations by the length of the elongation zone, i.e., up to 6*D* from the tip, in all experiments except for the ones where we specifically investigate the effects of a shorter elongation zone of 3*D*. For a relatively small time step of Δ*t* = 0.1 hour (as used in our simulations) and the average root growth rate of 1*D*/hour, we get $n=\frac{6.0}{0.1*1.0}=60$ 3-dimensional sections of the initial root. This way, we create segments having $\frac{D}{10}$ length. If the elongation region is shorter, then a smaller number of sections will be used. In our implementation, we keep incremental arrays to trace center positions and the basis for each segment.

From these arrays, we can easily draw the root by projecting the circumference points at |*r*(*d*)| on the *xy* plane from the center for each segment and use the mesh grid to connect consecutive circles.

The process starts from the 60^th^ segment at *d* = 6.0*D*, where it is calculated the growth response surface *G*_*s*_(*t*,*d*,*ϕ*) (as described below). If the provided elongation is differential (i.e., with greater values for some angle *ϕ*), then the first section will not only extend but also bend. Since the 59^th^ section is attached to the 60^th^ section, 3-D positions and orientations of all sections starting from the 59^th^ will be affected according to the final orientation and position of the 60^th^ section. Then, we repeat the same process for the 59^th^ section by applying the aggregated growth response surface data given for *d* = 5.9*D*. And so forth for 58^th^ and following sections. Iteratively, we extend and bend all 60 sections. The total length of these 60 sections will now be greater than the initial length of 6.0D. Since the length of the elongation zone is always fixed to 6.0*D*, the next iteration will again consider 60 sections of 0.1*D* length each. When going from 60 elongated sections to 60 sections of length 0.1, we preserve their 3-D orientation. The region above the 6.0*D* does not move and is no longer updated.

The orientation of the coordinate system in the first section (at *d* = 6.0*D*) at initial time is defined by the initial orientation of the root. The kinematics of the root can be described by roto-translation matrices, accounting for root extensions and bending, applied to update the basis $A(t,d)\in R^{3\times 3}$ build for each segment at time *t* and distance *d* from the tip (frame *Ox*^0^*y*^0^*z*^0^ in the segment shown as an example in Fig 1C). Each next cross-section is orientated w.r.t. the basis of the previous cross-section and any 3-dimensional bending or growth of the root that needs to be applied at each time step iteratively affect all subsequent sections.

For each time step, once *G*_*s*_(*t*,*d*,*ϕ*) is obtained for all *d* (center distance of the segment) and *ϕ* (with *ϕ* step of 360°/361° in our implementation), we can extract the *G*_*left*_ as the maximal rate of growth with its corresponding angle *ϕ*, and *G*_*right*_ as the rate of growth at *ϕ*+*π*. The bending angle can then be derived as in Eq (1) with a curvature radius equal to:

$$R_{c}=\frac{D}{2}\frac{G_{left}+G_{right}}{G_{left}-G_{right}}$$ (3)

To define the new basis *A*(*t*+Δ*t*, *d*), we need to define the proper rotation matrix ($M\in R^{3\times 3}$) to be applied and the center of the new frame. Building the frame *Ox*′*y*′*z*′ (Fig 1D) with center (0 0 0) (Fig 1), we rotate the line $\bar{u^{\prime }}=\left(\begin{array}{ccc} 0 & 0 & 0\\ 0 & -R_{c} & 0 \end{array}\right)$ and *z*′ by the angle *θ* around the *x*′ axis with the rotation matrix:

$$R=\left(\begin{array}{ccc} C_{\theta }+{{x^{\prime }}_{x}}^{2}(1-C_{\theta }) & {x^{\prime }}_{y}{x^{\prime }}_{x}(1-C_{\theta })-{x^{\prime }}_{z}S_{\theta } & {x^{\prime }}_{z}{x^{\prime }}_{x}(1-C_{\theta })+{x^{\prime }}_{y}S_{\theta }\\ {x^{\prime }}_{y}{x^{\prime }}_{x}(1-C_{\theta })+{x^{\prime }}_{z}S_{\theta } & C_{\theta }+{{x^{\prime }}_{y}}^{2}(1-C_{\theta }) & {x^{\prime }}_{z}{x^{\prime }}_{y}(1-C_{\theta })-{x^{\prime }}_{x}S_{\theta }\\ {x^{\prime }}_{z}{x^{\prime }}_{x}(1-C_{\theta })-{x^{\prime }}_{y}S_{\theta } & {x^{\prime }}_{z}{x^{\prime }}_{y}(1-C_{\theta })+{x^{\prime }}_{x}S_{\theta } & C_{\theta }+{{x^{\prime }}_{z}}^{2}(1-C_{\theta }) \end{array}\right)$$ (4)

where *C*_*θ*_ denotes cos *θ* and *S*_*θ*_ denotes sin *θ*, obtaining $\bar{u^{\prime \prime }}=\bar{u^{\prime }}R$, $\vec{z^{\prime }}=\vec{z^{\prime }}R$ and $\vec{v}=(\begin{array}{ccc} {\bar{u^{\prime \prime }}}_{11} & {\bar{u^{\prime \prime }}}_{12} & {\bar{u^{\prime \prime }}}_{13} \end{array})$. Translating $\vec{u^{\prime \prime }}+\left(\begin{array}{ccc} 0 & R_{c} & 0\\ 0 & R_{c} & 0 \end{array}\right)$ and normalizing the resulting vector we get *y*′′. With the cross product *x*′′ = *y*′′×*z*′′ we have a new basis *Ox*′′*y*′′*z*′′. Here, we have to apply a rotation by *ϕ* about *z*′′ to obtain the rotation matrix *M*:

$$M=\left(\begin{array}{ccc} C_{\phi } & -S_{\phi } & 0\\ S_{\phi } & C_{\phi } & 0\\ 0 & 0 & 1 \end{array}\right)(x^{\prime \prime }\;y^{\prime \prime }\;z^{\prime \prime })$$ (5)

At time *t*+Δ*t* we can apply *A*(*t*+Δ*t*,*d*) = *A*(*t*,*d*)∙*M* to obtain the new frame *Oxyz* for the segment at distance *d* in the root, having the center in $A(t,d)∙{\vec{v}}^{\prime }$. By controlling *G*_*s*_(*t*,*d*,*ϕ*) over time, any 3-D trajectory can be generated and, thus, any single root apex’s behavior. Three examples of stimuli growth response surfaces *G*_*s*_(*t*,*d*,*ϕ*) with corresponding root trajectories are shown in S1B, S1E and S1H Fig.

We hypothesized that *G*_*s*_(*t*,*d*,*ϕ*) can be decoupled into two terms: i) a slower changing term called the *baseline growth response surface G*_*b*_(*t*,*d*,*ϕ*), which defines root zonation, mainly determined by genetic actors, and ii) a faster changing term associated with the *aggregated signal response surface S*_*a*_(*t*,*d*,*ϕ*) which defines the growth excitation or inhibition according to incoming and previously received signals. More formally, the stimuli growth response surface is obtained as:

$$G_{s}(t,d,\phi )=G_{b}(t,d,\phi )∙S_{a}(t,d,\phi ),$$ (6)

where *G*_*b*_(*t*,*d*,*ϕ*) provides the genetically defined pattern of growth as a function of the distance from the tip, also described as root zonation. While recent observations suggest the influence of internal and external resources on root zonation [50, 51], we kept constant this pattern of growth in our model. We described it with a Gaussian distribution with a standard deviation set to 0.1 times the length of the elongation region and peak in the middle of the elongation region (in S1B Fig, at *d* = 3*D*, the peak is at 56.06% of the elongation rate). *S*_*a*_(*t*,*d*,*ϕ*) represents the aggregation of *N* individual stimulus signal response surfaces, obtained as:

$$S_{a}(t,d,\phi )=\left(1-\frac{\Delta t}{\tau (t,d,\phi )}\right)∙S_{a}(t-\Delta t,d,\phi )+\frac{\Delta t}{\tau (t,d,\phi )}∙f_{a}(\sum_{i}^{N}w_{i}(t,d,\phi )S_{i}(t,d,\phi )+b(t,d,\phi )).$$ (7)

In Eq (7), the *S*_*i*_(*t*,*d*,*ϕ*) is the *i*-th stimulus signal response surface, *w*_*i*_(*t*,*d*,*ϕ*) is its *t* time- and (*d*,*ϕ*) position-dependent weight, *b*(*t*,*d*,*ϕ*) is a bias, *f*_*a*_ is an activation/aggregation function (that we assumed here to be linear *f*_*a*_(*x*) = *x*) and *τ*(*t*,*d*,*ϕ*) is the signal decay. Therefore, the aggregated signal response surface naturally depends on the sensitivity *w*_*i*_(*t*,*d*,*ϕ*) of a particular point (*d*,*ϕ*) at time *t* to incoming signals, aggregated with *f*_*a*_.

The *i*-th (out of *N*) stimulus signal response surface can be associated with a chemical regulator that we modeled with a signal that originates at some position in (*d*,*ϕ*)-space with strength *S*_*i*_(*t*)∈[−1,+1] (where −1 is an extreme inhibition and +1 is an extreme excitation) and axially propagated according to a velocity vector $\bar{v_{i}}$. *S*_*i*_(*t*,*d*,*ϕ*) = 0 is the initial condition that remains 0 if no signal is generated and propagated. Different generation functions can be drawn for the chemical regulator, according to the type of stimulus. In the following, we will detail the case of gravity signal generation (*S*_*g*_), thigmo-signal (*S*_*t*_) and internal oscillator signal (*S*_*s*_) functions; also presenting two possible conditions for *τ*(*t*,*d*,*ϕ*) when discussing mechanical stimuli.

The sensitivity of particular regions to signals (e.g., regulated in nature by the auxin) could eventually change over time by changing *w*_*i*_(*t*,*d*,*ϕ*), possibly playing a role in the dynamic evolution of root zonation [50, 51]. Also, by controlling *b*(*t*,*d*,*ϕ*), a biased growth is achieved: by increasing or decreasing the bias, more excitation or inhibition is obtained in the corresponding region (*d*,*ϕ*)-space over time *t*, possibly helping describing natural or salt-induced asymmetric developments observable in plants [52–54]. In our simulations, we set *b*(*t*,*d*,*ϕ*) at 1 in order to have *G*_*s*_(*t*,*d*,*ϕ*) equal to *G*_*b*_(*t*,*d*,*ϕ*) in case of no signals, and we imposed *w*_*i*_(*t*,*d*,*ϕ*) to be constant over time and space for all simulations.

As a consequence, the patterns of motion in the roots by our model highly depends on the sensitivities *w*_*i*_(*t*,*d*,*ϕ*) to incoming signals, signals decay, and root zonation. Examples of model parameterization are given in the S1 File and S1 Video. Table 1 in S1 File summarizes the parameters settings adopted for each simulation performed in this study.

It is worth noticing that the formalization of our root growth model (Eq (7)) is analogous to the model of a continuous-time recurrent neural network [55, 56], which can formally be described as:

$$\tau_{i}\dot{y_{i}}={-y}_{i}+f_{a}(\sum_{j}^{N}w_{ji}y_{j}+b_{i})+I_{i}(t),$$ (8)

and resolved by the Euler step method for *i*-th neuron, where *y*_*i*_ is an activation of the postsynaptic node (in analogy, *S*_*a*_(*t*,*d*,*ϕ*) in Eq (7)), $\dot{y_{i}}$ is a rate of an activation change of the postsynaptic node (((*S*_*a*_(*t*,*d*,*ϕ*)−*S*_*a*_(*t*−Δ*t*,*d*,*ϕ*))/Δ*t* in Eq (7)), *τ*_*i*_ is a time constant of the postsynaptic node (*τ*(*t*,*d*,*ϕ*) in Eq (7)), *f*_*a*_ is an activation function (often sigmoid), *b*_*i*_ is a bias of the presynaptic node (*b*(*t*,*d*,*ϕ*) in Eq (7)), and *I*_*i*_(*t*) is an input (if any) to node *i* (not present in Eq (7)). By this formalization, the root is acting as an artificial neural network where signal velocity transmission and nodes sensitivity to signals define the final root growth patterns.

### Modeling of gravitropism

**Fig 2:**
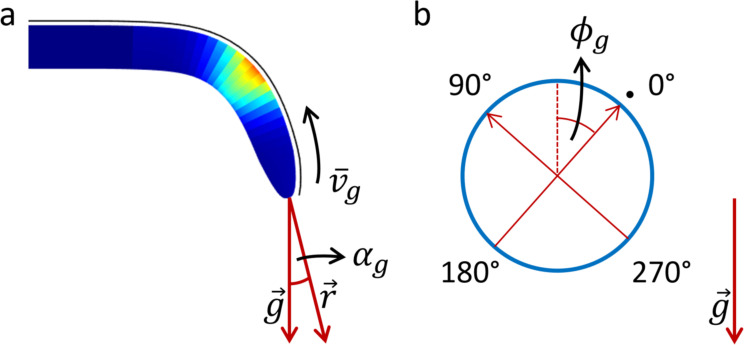
Schematic depicting key parameters under gravitropic signal. (a) A bending root responding to gravity, showing the root tip orientation $\vec{r}$, the gravity vector $\vec{g}$, the angle *α*_*g*_ gives the deviation of the root from the gravity, and the signal velocity ${\bar{v}}_{g}$. (b) A cross-section of the root showing the angle *ϕ*_*g*_ in the circumference along which the maximal signal is propagated. The reference line is drawn in black in (a) and with a black dot in (b).

Gravitropic signals originate in the root cap (*d* = 0), often viewed as the center of gravity sensing [57], and propagate along the root with speed *v*_*g*_ (velocity ${\bar{v}}_{g}$ is set to be positive along the axis *d* and zero along *ϕ*). The minimal angle between the root’s orientation at the tip $\vec{r}$ (Fig 2) and the gravity vector $\vec{g}$ is defined by *α*_*g*_∈[0,*π*]. The strength of the signal (*S*_*i*_ in Eq (7), here with *i* = *g*) is obtained from *α*_*g*_ by:

$$s_{g}(t,0,\phi_{g})=\left\{ \begin{array}{c} \frac{{2\alpha }_{g}}{\pi },if\;\alpha_{g}\leq \frac{\pi }{2}\\ 1,otherwise \end{array}\right.,$$ (9)

such that it has its maximum *s*_*g*_(*t*,0,*ϕ*_*g*_) = 1 when *α*_*g*_ = *π*/2 (meaning, the tip is perpendicular to the gravity vector), while *α*_*g*_ = 0 (the tip is aligned with the gravity vector) corresponds to *s*_*g*_(*t*,0,*ϕ*_*g*_) = 0. The form of the signal is set to be symmetrical such that *s*_*g*_(*t*,0,*ϕ*_*g*_±*π*) = −*s*_*g*_(*t*,0,*ϕ*_*g*_), with *ϕ*_*g*_ representing the angle along the root circumference corresponding to the top side of the root (Fig 2), and the scaling between *ϕ*_*g*_ and *ϕ*_*g*_±*π* is linear. This signal behavior could be associated with a gravity-induced redistribution of signal carriers such as auxin. The speed of signal carriers *v*_*g*_ defines how long it will take to propagate to specific growth regions of the root. Furthermore, each surface element of the root, depending on its spatial location (*d*,*ϕ*), may have its own sensitivity/weight *w*_*g*_(*t*,*d*,*ϕ*) which contributes (here, *f*_*a*_(*x*) = *x*) to the aggregated stimuli signal surface (Eq (7)) and the stimuli growth response surface (Eq (6)).

### Modeling of thigmotropism

**Fig 3:**
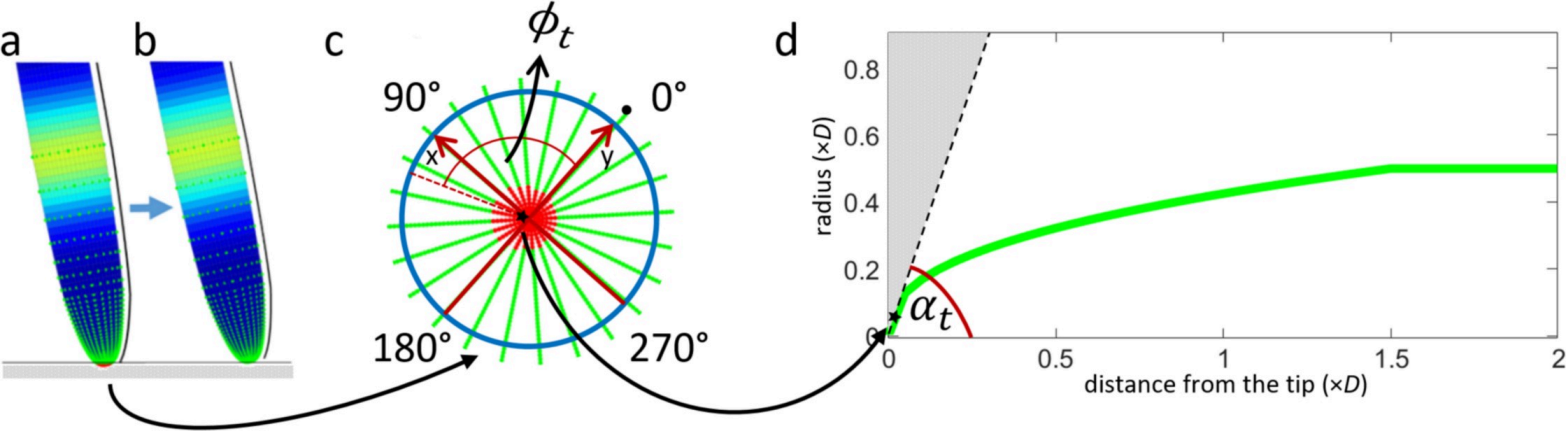
Modeling of thigmotropism. (a) An example of root apex intersecting with an obstacle depicted by a horizontal gray line. The root is covered with tactile sensory points, colored in green when no intersection is detected and colored in red when an intersection is detected (e.g., here at the very tip). A black line depicts the reference curve. (b) The corrected root (a) after a minimalistic bending to remove the intersection with the obstacle. (c) Tactile sensory points, shown from the bottom (tip) view on the root, are equidistantly spaced w.r.t. the center of the root in this view. The points are located at different angles *ϕ* w.r.t. the reference curve (*ϕ* = 0, black dot) depicted by the black line in (a, b). The mean of the intersected (red) points depicted by a black star represents an estimated location of the occurred touch. (d) The root shape is defined by a green line which illustrates how the root (*y*-axis) radius changes as a function of the distance from the tip (*x*-axis). *x* and *y* represent the multiplier for *D* as unitary value. Knowing the position of the mean of the activated sensory points (black star) is sufficient to estimate the *α*_*t*_ angle w.r.t. the obstacle (represented in the figure with the light gray triangle) as the angle between the centerline of the root and the tangent (dashed black line) at the starred point.

Thigmotropism, the root’s ability to respond to touch stimuli, is modeled firstly as a passive mechanical response which may require the root to bend when pushing against an obstacle and, secondly, as an active response which can be viewed as a continuation of the passive one and leads to a propagation of a touch signal *s*_*t*_. To estimate the region of touch and initiate the corresponding signal, the root is covered with sensory points (green points in Fig 3A and 3B). During numerical simulations, these points may intersect with obstacles that lead to detecting the intersection (intersected sensory points are shown as red points in Fig 3A and 3C) and produce a passive thigmotropic response (see Results section).

Specifically, from the set of sensory points that result in the intersection between root and obstacle (red points in Fig 3A and 3C), we estimate the mean point of touch as the vector sum of the intersected sensory points. From the mean point, having a certain distance *d* from the tip, and the associated angle *ϕ*_*t*_ w.r.t. the reference curve (see Fig 3C), we can calculate the radius *r*_*t*_ of the corresponding cross-section as in Eq (3). The mean point is depicted by a black star in Fig 3C and 3D and is associated with a particular angle *α*_*t*_ (Fig 3D) such that the points which are more distant from the very tip correspond to smaller values. Knowing the position of the mean of touched/intersected sensory points is sufficient to compute *α*_*t*_ as follows:

$$\alpha_{t}=\arctan\left(p_{cap}r_{t}^{p_{cap}-1}\right).$$ (10)

The strength of the signal linearly changes from *s*_*t*_ = −2*α*_*t*_/*π* at *ϕ* = *ϕ*_*t*_ to −*s*_*t*_ while approaching the opposite side of the root at *ϕ* = 2*π*+*ϕ*_*t*_. For the sake of simplicity, the symmetrical axial distribution of *s*_*t*_ w.r.t. *ϕ*_*t*_ mentioned above is assumed in the rest of the paper.

The modeling of passive thigmotropic response consists of iteratively performing a minimal and uniform bending, towards the very tip direction, of each modeled section of the root at *ϕ* = *ϕ*_*t*_ until the root stops intersecting with obstacles.

### Modeling of mechanically induced signaling memory

We investigated the conditions for the appearance of circumnutation movements in root-obstacle interactions. Root-obstacle interactions might mechanically-disturb cells, their sensitivity defined in this model by *w*_*t*_(*t*,*d*,*ϕ*), and their ability (e.g., the signal velocity vector $\bar{v_{i}}$) to pass signal-carriers. In our approach, we study the effect of root-obstacle interactions already at the point of their perception, i.e., at the mapping of the obstacle angle *α*_*t*_ to the inhibitory signal *s*_*t*_(*t*) as $s_{t}(t)=\left(1-\frac{\Delta t}{\tau (t,d,\phi )}\right)(t-\Delta t)+\frac{\Delta t}{\tau (t,d,\phi )}(-2\alpha_{t}(t)/\pi ).$. We analyzed two different conditions for the signal decay (how gradual the mapping/perception is), one with *τ*(*t*,*d*,*ϕ*) = Δ*t* (immediate initiation of the inhibition, giving *s*_*t*_(*t*) = −2*α*_*t*_/*π* as before) if the touch occurs, and the other case where the inhibitory signal *s*_*t*_(*t*) decays at a slower rate with *τ*(*t*,*d*,*ϕ*) = 10 hours. The slower rate leads to delayed signaling, which takes into account the memory of recent root-obstacle interactions.

### Modeling circumnutation movements for resources exploration

We investigated the possibility that plant roots perform circumnutations to explore environmental resources. In this case, we hypothesize the existence of an internal oscillator that generates a sinusoidal signal. This hypothesis seems to be supported by recent investigations suggesting the presence of an auxin-meditated signal, directionally transported, that regulates root cellular elongation and producing circumnutation [58]. This internal oscillatory apparatus is affected by the gradient of resources perceived at time *t* $\Delta C=\frac{C(t)-C(t-\Delta t)}{\Delta tG_{avr}(t)}$ [59], obtained as the difference of the concentrations perceived at current *C*(*t*) and previous time step *C*(*t*−Δ*t*) normalized on the average growth for the time step Δt*G*_*avr*_(*t*). This oscillator produces a signal at the tip as follows:

$$s_{s}(t,d=0,\phi =0)=\sin(t_{s}(t)),$$ (11)

$$t_{s}(t)=t_{s}(t-\Delta t)+\Delta tT_{s}(1+\frac{sign(\Delta C)}{1+exp|\Delta C|}),$$ (12)

where *t*_*s*_(*t*) defines the time of the internal oscillator (as a function of the elapsed time *t* of simulation, with *t*_*s*_(0) = 0 whose rate of change Δ*tT*_*s*_ for a frequency $T_{s}=\frac{2\pi }{P}>0$ (with *P* time in hour) can be accelerated (decelerated) by a factor of $\left|\frac{1}{1+exp|\Delta C|}\right|$ if the concentration increases as Δ*C*>0 (decreases as Δ*C*<0). Alternative functional forms of Eq (12) can be used as long as greater values of Δ*C* lead to greater positives changes of *t*_*s*_(*t*). The proposed function has a discontinuity when Δ*C* = 0, making the oscillator sensitive primarily to the sign and only secondarily to the amplitude of this change. The control of *s*_*s*_(*t*,*d*,*ϕ*) in 2-D space limits to *ϕ* = 0 and *ϕ* = ±π, whereas growth control in 3-D space can take any *ϕ*∈[−π,π] according to the frequency of oscillation as *ϕ*(t) = π+*T*_*s*_t. An example of a 3-D growth is shown in S1G Fig, where oscillatory signals originate at the tip, at a moving ${\phi (t)=\frac{\pi }{2}+\pi t/10}_{}$ for *t* = 0,…,10 hours, having *P* = 10 hours, that leads to a 3-D helical growth as shown in S1I Fig. The above-described oscillatory apparatus is not unique, and alternative configurable oscillators might be considered. See S2 Fig for three examples of roots performing different circumnutations and the strategy of their quantification.

## Results and discussions

### Effects of gravity on circumnutations

Among the several behavioral responses to environmental stimuli, gravitropism is the ability of plant roots to adjust their growth direction to align with the gravity vector by bending downwards when tilted perpendicularly with respect to gravity [60]. Overshooting of the gravity vector was suggested to be the origin of circumnutation movements [61] until a study demonstrated that circumnutations continue despite the absence of gravity [62], although the same research suggested, however, that gravity could affect circumnutations. The relation between root zonation and gravitropism response has been thoroughly analyzed, allowing, for instance, to define the differential growth rate, the minimum stimulation time, and time and region of stronger response translated in a higher differential growth [63, 64]. This region will also be called in this paper root zonation peak (see S1B, S1E and S1H Fig). Here, we studied the role of zonation and gravitropism in circumnutation movements by positioning the simulated root horizontally respect to the gravity and allowing it to grow. In particular, we investigated the effects of the propagation speed of gravitropic signals defined by *v*_*g*_, the root sensitivity to gravitropic signals defined by *w*_*g*_(*t*,*d*,*ϕ*) over time *t* and space (*d*,*ϕ*), and the zonation defined by *G*_*b*_(*t*,*d*,*ϕ*) on the growth behavior (see Materials and Methods for implementation). A first group of nine experiments corresponded to the case of a root zonation peak located at a distance from the tip of 1.5 times the averaged diameter of the formed root (*d* = 1.5*D*), whereas a second group of nine experiments corresponded to the case of a root zonation peak located at *d* = 3.0*D* from the tip.

**Fig 4:**
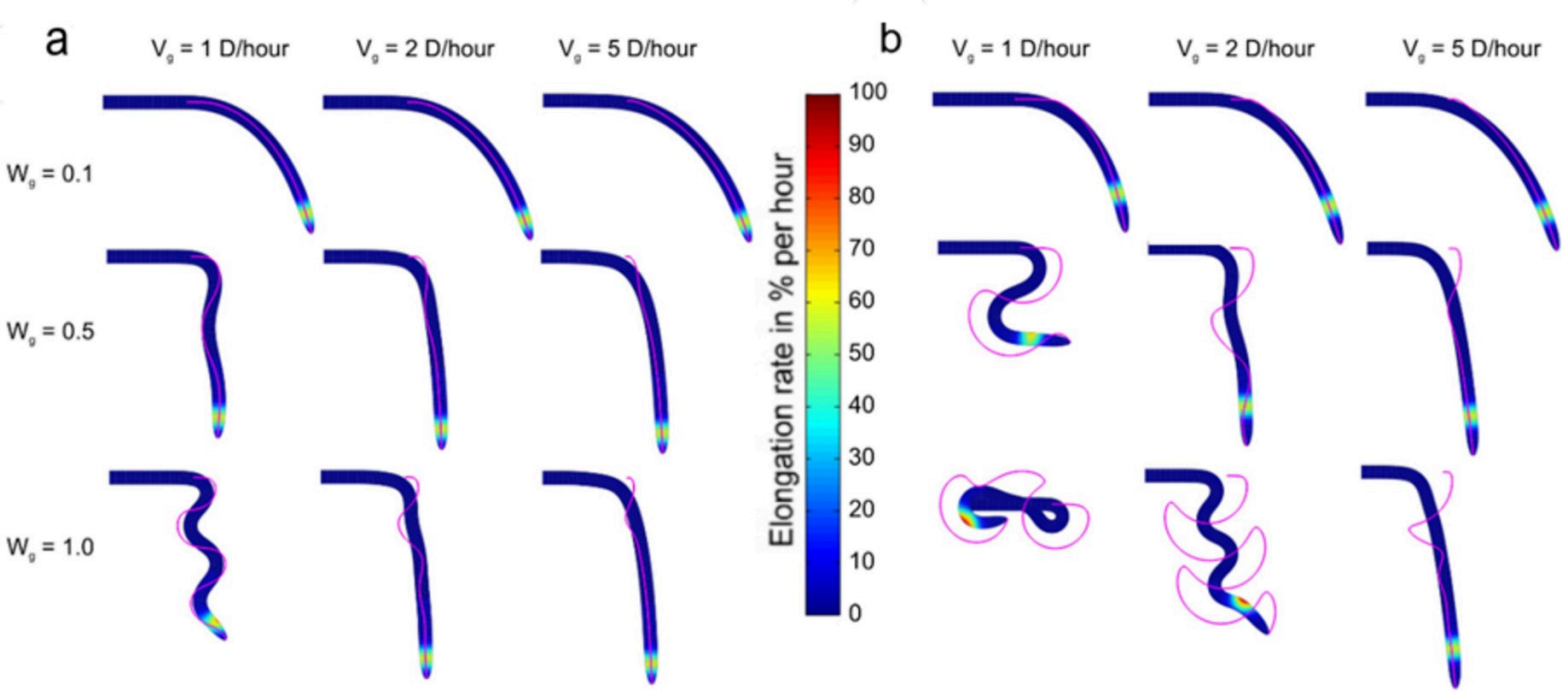
Effects of signal speed, sensitivity, and zonation on root growth. Two different root zonation peaks are compared (in colored scale, where red represents the location of maximum response): (a) the peak of the growth rate (%) is located at a distance of 1.5 times the average diameter of the formed root (*d* = 1.5*D*) from the tip; (b) the peak of the growth rate (%) is located at a distance of *d* = 3.0*D* from the tip. All the roots present the result after 16 h of gravitropic stimulations with a different propagation speed (*v*_*g*_) and sensitivity (*w*_*g*_). Magenta lines depict the trajectories of the tip. Colors show the rate (%) of elongation per h.

Both the gravitropic signal speed *v*_*g*_ and the root sensitivity to gravity *w*_*g*_ affected the root’s ability to converge to a stable vertical orientation (Fig 4 and S2 Video). Weak sensitivity *w*_*g*_ led to a steady downward bending without circumnutation movements, independently of the signal speed and location of the zonation peak. For zonation peaks closer to the tip (Fig 4A), we found that slower signal speeds (e.g., when the transport of signal carriers was not faster than 5 times the baseline root growth rate) and higher sensitivity (e.g., characterized by a substantial difference in concentrations of the auxin on the opposite sides of the root, which in turn caused differential growth [65]) generated circumnutation movements with different periods and amplitudes. We also found circumnutation movements to be more pronounced for zonation peaks at longer distances from the tip (Fig 4B); only a few simulated roots were unable to follow gravity and self-collided even considering a strong sensitivity (*w*_*g*_ = 1.0 for all possible values of (*d*,*ϕ*)), e.g., the case where signal speed was slower (*v*_*g*_ = 1*D*/*hour*) and zonation was farthest (*d* = 3.0*D*) (the self-collision check was intentionally disabled in simulation). These observations suggest that circumnutation movements can ultimately originate in response to gravistimulation. A detailed analysis of the impact of root zonation on gravitropism is provided in S3 Fig. Moreover, with our model, a greater sensitivity to gravity (high *w*_*g*_) led to more circumnutation movements for every signal speed (i.e., any column in Fig 4). This observation can be compared to a large-scale study in living plants [60], which showed that the youngest gravity-stressed seedlings of *Arabidopsis thaliana* tended to overshoot the gravity vector and initiate circumnutation movements. This way, our numerical experiments suggest that the youngest roots (as observed in [60]) might overshoot more than the older roots because they are more sensitive to gravity (behavior obtained with a greater value of *w*_*g*_ in our simulations, Fig 4). Besides, the diversity of root behavior obtained in simulation by setting different conditions (observable from the magenta lines in Fig 4 depicting the tip traces) matches with the diversity observed in the path, period, and amplitude, among different plants [66] or within the life of the same plant [67, 68]; thus suggesting that this variety of responses in plants may be induced by different signal propagation velocity, sensitivity and root zonation that might change according to the internal state of the plant (e.g., age, nutritional status, stress conditions) [66, 69–71] or signal pathways [72, 73]. We note that the speed of signal propagation *v*_*g*_ = 5D/hour and the average root growth rate of 1D/hour leads to a difference of a factor of 5. That factor corresponds to the center of the cluster of the auxin speeds and growth rates reported for 44 different species [74].

### How gravity and mechanical stimuli interact and affect circumnutations

**Fig 5:**
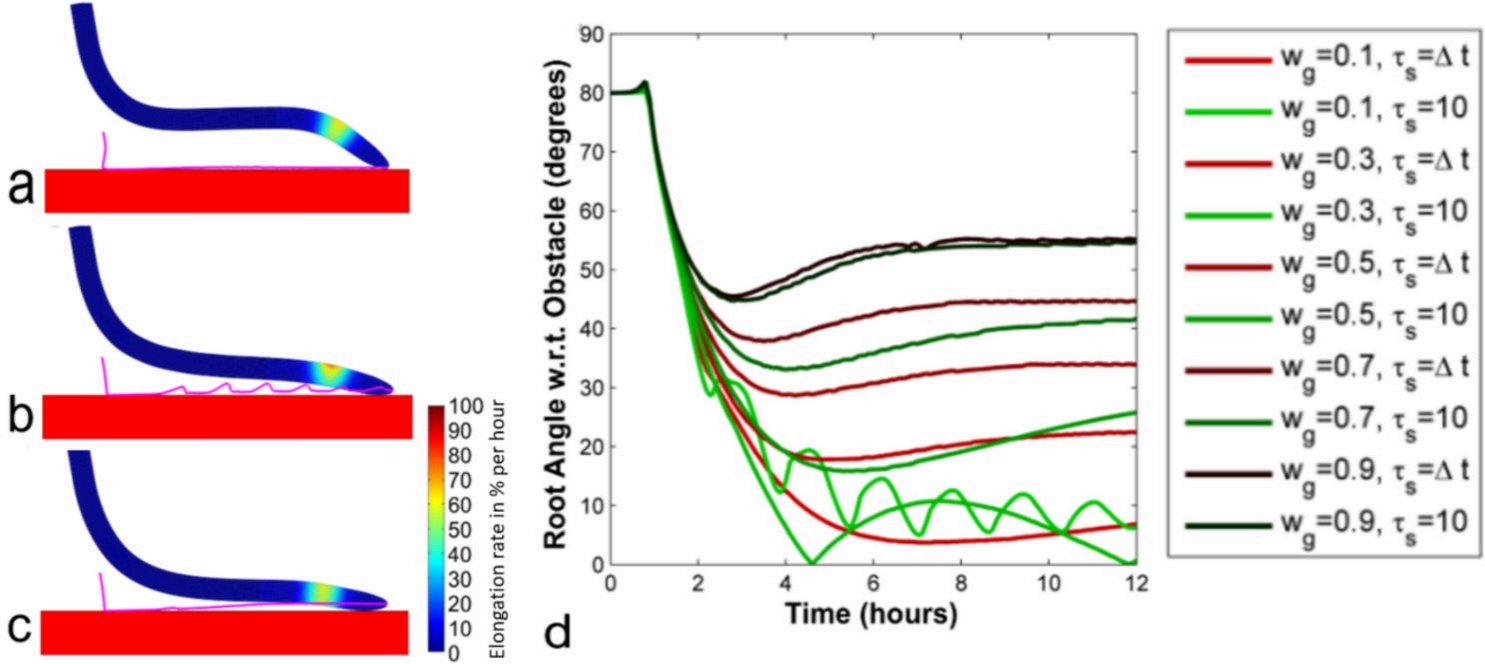
The interplay of gravity and touch responses. The sensitivity to gravitropic (*w*_*g*_) and thigmotropic (*w*_*t*_) signals was set at (a) 0.5 and 0.5, (b) 0.1 and 0.9, and (c) 0.9 and 0.1, respectively. Colors show the rate (%) of elongation per h. Magenta lines depict the trajectories of the tip. (d) Root angle w.r.t obstacle over time for roots with different *w*_*g*_ and *w*_*t*_ = 1−*w*_*g*_ for *τ*_*s*_ = Δ*t* (immediate update of touch signal) and *τ*_*s*_ = 10 (immediate increase but a slow decrease of touch signal).

In addition to gravity, touch stimulation represents another critical factor influencing root growth [12, 75]. Thigmotropism is the ability of plant roots to respond to mechanical stimuli such as contact with obstacles. It has been observed that when a descending root encounters a horizontal obstacle, it assumes a sigmoidal shape, and the tip slides against a barrier [76, 77]. Although both thigmotropism and gravitropism are believed to be involved in root-obstacle interactions [76, 77] that may result in circumnutation movements [78], the underlying mechanisms of their interplay remain poorly understood. Here, we investigated these mechanisms by simulating both a gravitropic signal, which made the root align with the gravity vector and a thigmotropic signal, which stimulated the growth process in the area where the touch stimulus occurred. Simulated roots can contact obstacles (including other roots) but cannot penetrate them. The simulated root (Fig 5A and 5B and S3 Video) displayed a sliding behavior analogous to that of living roots [76, 77]. When sensitivities to gravitropic and thigmotropic signals were the same (*w*_*g*_ = *w*_*t*_ = 0.5), the tip of the root maintained an angle of approximately 45° while sliding against the surface (Fig 5A, and see S4 Fig for a 3D case). Different values of sensitivity to gravity *w*_*g*_ led to different angles (Fig 5D); for instance, relatively small values of *w*_*g*_ (which approximates weak sensitivity to auxin) led to small angles (Fig 5C and 5D). These results suggest that, since environmental cues affect plant hormone levels and responses over time (see [15] for a review), the angle observed for a given plant root can be viewed as a proxy to identify its current sensitivity to gravitropic and thigmotropic signals. These results also suggest that the formation of a sigmoidal shape along the root apex (the curvature developed in opposite directions in two regions [76, 77] is caused by the interplay of gravitropic and thigmotropic responses and may not require other specific control mechanisms. Investigations on different signal decays (*τ*_*s*_ = Δ*t* and *τ*_*s*_ = 10 hours) do not show relevant difference in root-to-obstacle angle (Fig 5D) and root growth behavior when the sensitivity to gravitropic signal (*w*_*g*_) is high. Whereas, with low *w*_*g*_, our numerical experiments show that root-obstacle interactions result in circumnutation movements [78] when the inhibitory signal resulting from touch raises much faster than it decays. An example of this root growth behavior is shown in Fig 5B (case with *w*_*g*_ = 0.1, *w*_*t*_ = 0.9, *τ*_*s*_ = 10), where the root performs circumnutation movements characterized by a periodic touch of the obstacle and a loss of contact with it (the tip trajectory is shown in magenta line). Such behavior suggests the presence of a mechanically induced signaling memory driving the movements. In certain environmental conditions (e.g., highly heterogeneous soils), these movements might represent a useful response to avoid obstacles instead of buckling against them.

### The role of a possible internal oscillator driving circumnutations

**Fig 6:**
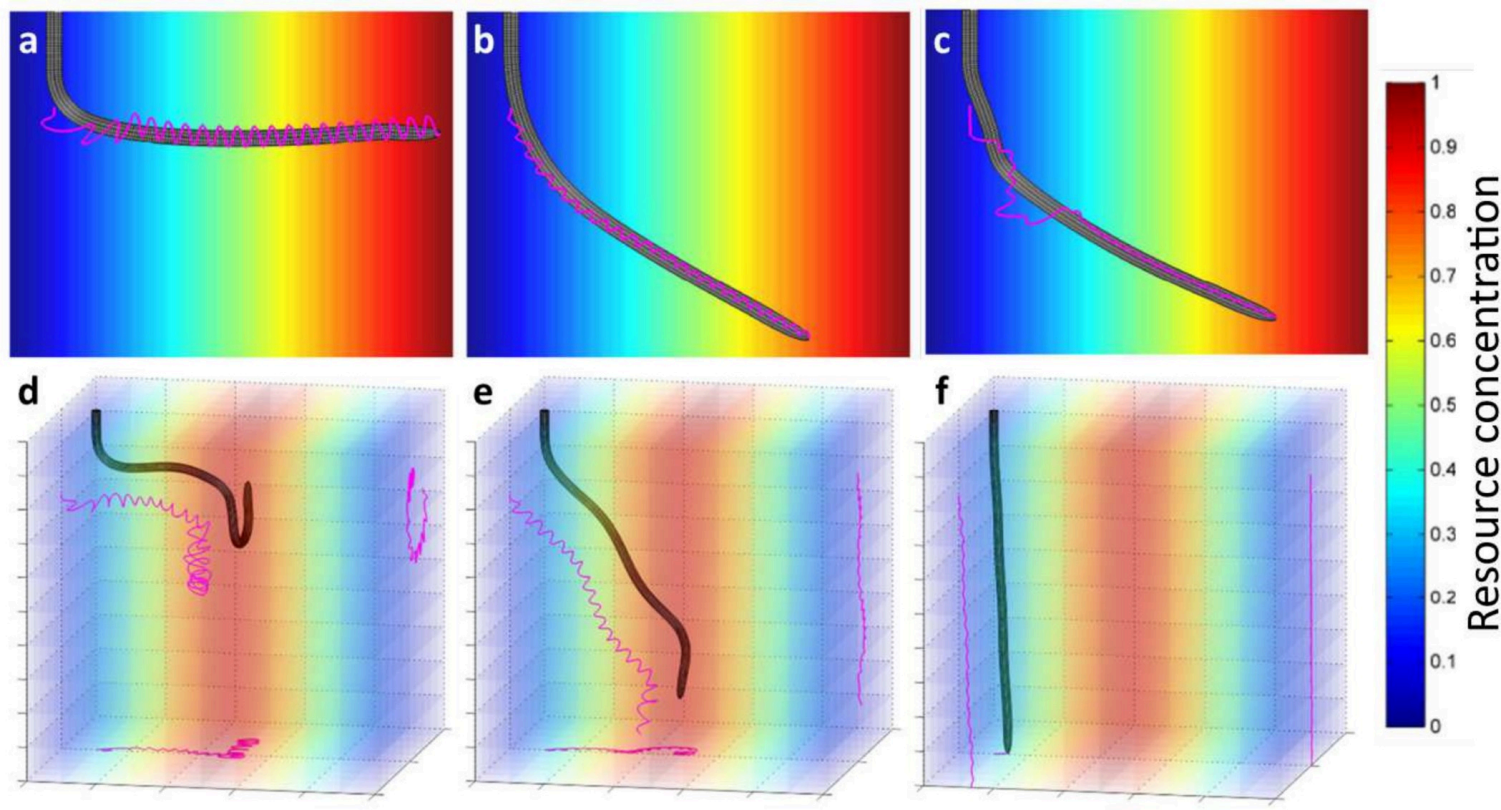
Roots navigate towards regions with higher resource concentration with circumnutation movements driven by an internal oscillatory apparatus. The resource concentration ranges from 0 (blue color) and 1 (red color). In the rows are shown the roots navigation (a, b, c) in the presence of only the resource signal active (*w*_*g*_ = *w*_*t*_ = 0, *w*_*s*_≠0) and (d, e, f) as a result of the interplay between gravity and resource stimulus responses (*w*_*t*_ = 0, *w*_*g*_≠0, *w*_*s*_≠0). (a) A sample of navigation with root settings *w*_*s*_ = 1, *v*_*s*_ = 5D/hour, *T*_*s*_ = 0.75*π*, *d* = 3.0*D*. (b) The path for a root with settings *w*_*s*_ = 0.25, *v*_*s*_ = 10D/hour, *T*_*s*_ = 0.94*π*, *d* = 2.0*D*. (c) A sample of a non-regular pattern of circumnutation movements obtained with root settings *w*_*s*_ = 0.75, *v*_*s*_ = 2.5 D/hour, *T*_*s*_ = 1.07 *π*, *d* = 4.5*D*. For simulations in (d, e, f), stimulus and gravity signals originate at the tip and propagate along the root with speed *v*_*s*_ = *v*_*g*_ = 5D/hour. The internal oscillator outputs *s*_*s*_ = 1 at *d* = 0 and *ϕ* which changes over time with frequency *T*_*s*_ = *π*. Weights of resources and gravity for each root were: (d) *w*_*s*_ = 0.9, *w*_*g*_ = 0.1; (e) *w*_*s*_ = 0.6, *w*_*g*_ = 0.4; (f) *w*_*s*_ = 0.1, *w*_*g*_ = 0.9.

Here, we hypothesize that, all other conditions being equal, circumnutation movements might be adaptive movements initiated by a root endogenous control system to estimate gradient information in the soil (e.g., water, salt, or temperature). This hypothesis, which we call the Information Hypothesis, is driven by the fact that sensors for soil parameters are concentrated at the root tip [79, 80] and may not accurately detect small gradient differences and orient the root growth appropriately. Indeed, other biological systems with similar sensory constraints, such as worms, display a similar waving behavior employing an internally dictated continuous oscillation of the head, adopted by the animals for comparing nutrient concentration over time and space in the environment while moving forward [59]. From this analogy and following the suggestion of Darwin and Darwin [81], we considered the presence of an internal oscillatory apparatus which the root would use for resource exploration. This apparatus generates a sinusoidal signal (Eq (11)) that controls the root growth as a function of a resource *C* defined as the perceived concentration of environmental resources (salt, water, or temperature, e.g.) according to the root preferences at time *t*. In contrast to our thigmotropic simulations, we do not model individual resource concentration sensors but consider that the concentration of the resource is measured at the very root tip as the aggregate measure of multiple, spatially-close sensors of the root tip. We performed 1,000 simulations using vertically oriented roots in a 2-D environment with a linear horizontal gradient of *C* (the gradient is built with a slope of 0.05 for every horizontal displacement of 1D, with a minimum of 0 and a maximum of 1, Fig 6A–6C and S4 Video) to evaluate the roots ability to increase the resource concentration measured at the tip after 30 h. We investigated different variants for the internal oscillatory apparatus with random settings of signal weight, speed of the signal propagation, frequency of the internal oscillator, and distance from the elongation zone. The details of the corresponding values are given in S5 Fig.

The results show that the internal oscillatory apparatus is sufficient to make the root grow towards regions of higher resource concentrations with only one sensor at its tip (Fig 6A–6C). Furthermore, the results indicate that the period and amplitude of circumnutation movements (S5 Fig) depend on the increase or decrease of the resource concentrations perceived at the tip. Different variants for the internal oscillatory apparatus resulted in different capabilities to reach environmental regions with higher resource concentration (Fig 6A–6C). We also found that the internal oscillator allows roots to reach regions of higher resource concentrations in 3-D settings while also affected by gravity (Fig 6E). Fig 6D–6F show the interplay between resource exploration and gravity stimulus by varying the corresponding weights. When the root is parametrized to have a higher sensitivity to the resource stimulus than to gravity (Fig 6D), it reaches the region of the maximum *C* by performing helical circumnutation movements. Then, it tends to stay in this region, almost unaffected by gravity. The root with comparable sensitivities between stimulus and gravity (Fig 6E) also performed a helical path while preserving their tip on average oriented towards gravity. Instead, the root dominated by the gravitropic signal (f) grows downwards with a reduced amplitude of circumnutations and is only slightly biased towards better values of *C*. These experiments suggest that the sensitivities to different stimuli determine the growth patterns of plant roots by setting priorities in exploring the environment (examples of circumnutation movements in 3D are presented in S5 Video).

### How endogenous and exogenous signals act in root specialization

**Fig 7:**
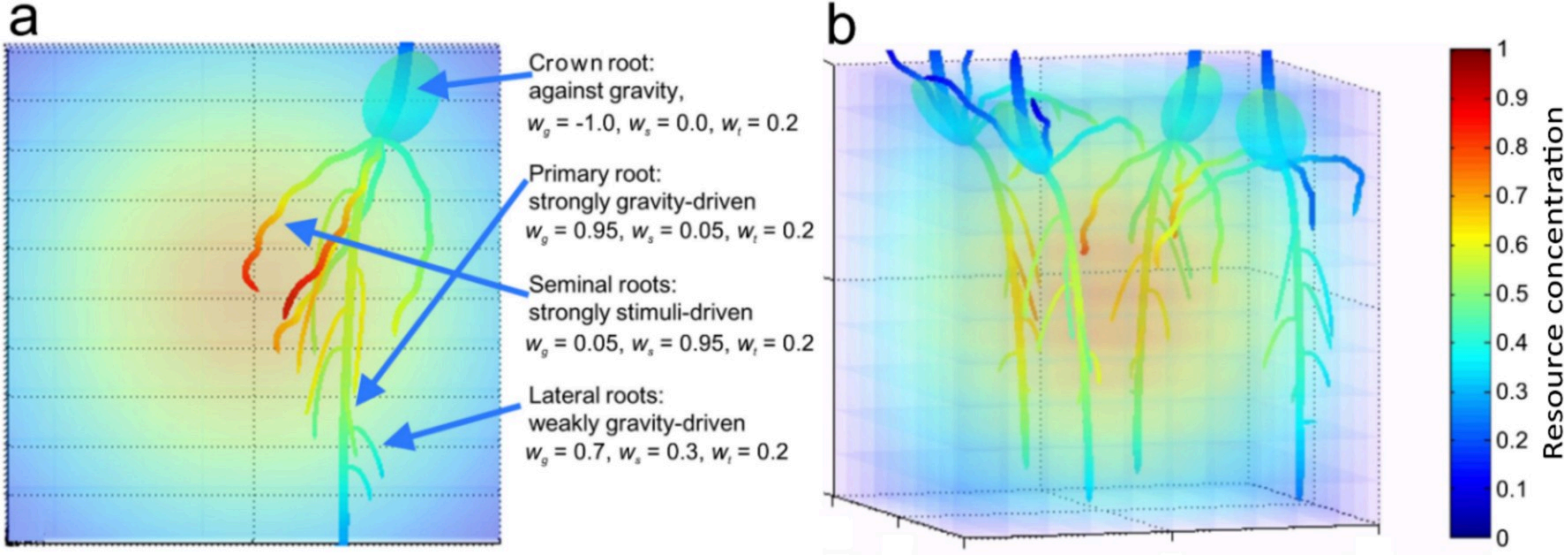
An example of root specialization. We investigated whether the complex ramification of the plant root systems could be approximated by the same relatively simple model for each root type, but with different root-specific (e.g., primary, secondary, crown roots) and task-specific (e.g., gravity-, resource-sensitive) parameter values. (a) Different roots (crown, primary, seminal and lateral roots) initialized with different sensitivity to gravitropic *w*_*g*_, thigmotropic *w*_*t*_, and resource *w*_*s*_ signals to very roughly approximate their possible behavior (e.g., the crown is initialized with negative gravitropism and upward growth by setting *w*_*g*_ = −1, seminal roots could be stimulated to circumnutate towards better environmental conditions barely affected by gravity when setting *w*_*s*_ = 0.7). All roots could avoid obstacles when setting *w*_*t*_ = 0.2. (b) Simulation of root specialization with parameters given in (a). Different kinds of roots were differently affected by the presence of the environmental resource, and the roots with greater *w*_*s*_ to the resource concentration *C* tended to grow towards the middle of the box where the maximum of *C* scaled between 0 (blue color) and 1 (red color).

Finally, we investigate whether the presence of the internal oscillator suggests that circumnutations should be permanent. To this aim, we used our model to describe and analyze growth movements and specialization of different root types (e.g., primary, secondary, crown roots) [82] within the same root system, accounting for an internal oscillator, by assuming that different root types have different sensitivities to environmental stimuli (e.g., gravity, resources). Such specialization has been indeed observed in natural roots [83], particularly in stress conditions [84–86]. We performed 3-D simulations with 4 different seeds (see Fig 7 and S6 Video), which can grow differently parameterize roots such as crown roots (*w*_*g*_ = −1, *w*_*s*_ = 0.0, *w*_*t*_ = 0.2), primary roots (*w*_*g*_ = 0.95, *w*_*s*_ = 0.05, *w*_*t*_ = 0.2), seminal roots (*w*_*g*_ = 0.05, *w*_*s*_ = 0.95, *w*_*t*_ = 0.2) and lateral roots (*w*_*g*_ = 0.7, *w*_*s*_ = 0.3, *w*_*t*_ = 0.2). The results showed that roots (here, primary roots) much more sensitive to gravity than environmental resources tend to grow downward unaffected by the internal oscillator. On the other hand, roots sensitive to environmental resources (here, seminal roots) employ circumnutation movements to accurately detect small resource gradient differences (in line with our Information Hypothesis) and orient the root growth towards better environmental conditions. Such behavior has not been shown yet in real roots in the presence of nutrient gradients. Nevertheless, inhibitory effects of one stimulus over another have been observed and described previously, like the case of salt over gravity [70] or humidity over gravity [79]. Other supporting studies have shown oscillatory movements of the root cap with a mean direction of growth towards the highest water potential [87], and the reduction of circumnutations in the presence of aluminum [70]. However, how root sensitivities are determined remains unclear, but they might result from the evolutionary adaptation of plant species to the environment.

## Conclusions

Circumnutation movements have been since long investigated and different hypotheses have been formulated to explain their existence. Darwin and Darwin [81] suggested that circumnutation movements are an endogenous process under plants’ internal control. In the 1960s, the focus shifted towards the gravity-based hypothesis [61], which suggested that gravity can cause overshooting and circumnutation movements. When experiments in outer space showed that gravity is not a necessary condition for the appearance of circumnutation movements [62], Darwin’s hypothesis regained popularity. The current consensus is that circumnutation movements have both endogenous and exogenous origin [66], but due to difficulties in observation and disambiguation of roots behavior, the purpose (if any) of these movements remains poorly understood.

Here, we propose a computational model to simulate growth patterns of plant roots given different internal and external conditions and analyze the emergence and the role of circumnutation movements. In the model, the surface of the root is viewed as covered by artificial neuron-like nodes sensitive to input signals and working as a continuous-time recurrent neural network, through which the signal is propagated along the growth region (defined here by root zonation), where the differential growth can be actuated as a response. The analogy with a time recurrent neural network is justified by the fact that the imprint of environmental stimuli throughout time defines the phenotype of the plant [88, 89], actuated by processes adapted through evolution that exhibit a sort of memory of the environmental changes throughout the plant lifetime [39, 88].

According to the several theories formulated about the origin of circumnutations, we investigate over multiple parameters, such as root zonation, signal sensitivity, and propagation speed, to identify the conditions for circumnuations to emerge in the presence of exogenous stimulation and an endogenous control system. Specifically, by using our computational model, we first investigate the effects of the single gravitropic signal, tactile stimulation by placing barriers in front of the growth pattern, and considering tactile and gravity signals in interaction. Then, we introduce an endogenous oscillatory control system.

Ultimately, the proposed computational model suggests that circumnutations can emerge a) as an overshooting of the gravity vector when gravitropic signals are slow w.r.t. the root growth rate, b) as a result of root-obstacle interactions when touch-mediated root inhibition response raises faster than it decays, c) as a result of an internal oscillator as predicted by Darwin and Darwin [81]. All other factors being equal, the latter finding suggests that circumnutations could play a functional role in detecting and reacting to environmental gradients.

## Supporting information

S1 FigExamples of root growth with a different parametrization of signal and growth response surfaces.(a) An aggregated signal response surface in time **t**, distance **d** from the tip along the center of the root normalized by **D**, and angle **ϕ** w.r.t. **r**, which is used as a reference curve (black lines in (c), (f), and (i)). The present aggregated signal response surface has no inhibited/excited regions. Colors in (a), (d), and (g) show the amplitude of the aggregated signal. (b) A baseline growth response surface defines the zones of faster/slower elongation. Colors in (b), (c), (e), (f), (h), and (i) show the rate (%) of elongation per h. (c) A straight root with the reference curve depicted by a black curve. Aggregated signal response (d) and growth response (e) surfaces after 2 h of excitation signals originating at (**d** = **0**, **ϕ** = **0**) and propagating along the root with a speed of 5**D** per h lead to a 2-D bending as shown in (f). The same excitation signals originated at **d** = **0**, but with a periodically changing angle **ϕ** in [−**π**,+**π**], as $\phi (t)=\frac{\pi }{2}+\frac{t\pi }{10}$ for **t** = **0**,…,**10h** (g, h) lead to a 3-D helical growth (i).(TIF)

S2 FigQuantification of circumnutation movements.(a, d, g) Three examples of roots performing circumnutation movements driven by an internal oscillatory apparatus. (b, e, h) The normalized growth rate on one side of the root versus time. The red points correspond to the minima and maxima growth rate, and, thus, to the maxima differential growth. The time difference between the red points is used to compute the periods of circumnutation movements. (c, f, i) The red points detected in (b, e, h) are translated into the original coordinate system. By taking any three consecutive red points ***p***_**1**_, ***p***_**2**_, ***p***_**3**_ it is possible to estimate the amplitude of one cycle of circumnutations as $\left\|\frac{p_{1}+p_{3}}{2}-p_{2}\right\|$, i.e., the distance between the second point and a mean position between the first and the third points.(TIF)

S3 Fig Effects of signaling parametrization on root gravitropism. The results in (a) and (b) show the root angle w.r.t. the gravity vector versus time for Fig 4A and 4B, respectively. (c) The mean elongation rates versus distance from the tip for (a) and (b) together with the median, first and third quartiles of differential elongation rates. The twice more distant location of the main growth zones in (b) compared to (a) statistically significant (Wilcoxon rank-sum test, ***p*** = **10**^**−9**^) by a factor of 1.68 increases the median amplitude of differential growth (c), the dynamic change of the latter causes circumnutations.
(TIF)

S4 Fig Modeling of thigmotropism. A 3-D example of root interacting with obstacles, with wg = 0.5, ***w***_***g***_ = **0.5**, ***w***_***t***_ = **0.5**. The projections of the root tip trajectories are depicted by magenta lines.
(TIF)

S5 FigLikelihood of parameters in selected groups.The results of 1,000 experiments with initially vertically oriented roots in a 2-D environment with a linear gradient of ***C***, as shown in Fig 6A–6C. The signaling apparatus of each root was parametrized with uniformly randomly generated stimulus signal weight ***w***_***s***_∈[**0, 1**], speed of signal propagation ***v***_***s***_∈[**0, 10**]D/hour, baseline oscillator frequency ***T***_***s***_∈[**0,2*π***], location of the peak of elongation ***d***∈[**0.5, 5.5**]***D***. The performance of each root was measured after 30 simulated hours as $f=\frac{C(30)-C(0)}{C^{*}(30)-C(0)}\in [-1,1]$ w.r.t. the best possible C*(**30**). Ranked after ***f***, 5 non-exclusive groups were selected: ***G***_**1**_ with best 5% roots and median ***f*** = 0.8156, ***G***_**2**_ with best 25% and ***f*** = 0.6925, ***G***_**3**_ with worst 25% and ***f*** = -0.524, ***G***_**4**_ with worst 5% and ***f*** = -0.706, and ***G***_**5**_ with all roots and ***f*** = 0.0671. (a-d) The likelihood (measured by kernel density estimation as in [89]) of each group to have specific parameter settings. The best performing roots (group ***G***_**1**_, an example is given in Fig 3A) are more likely to have stronger sensitivity to stimulus (see a), faster speed of signal propagation (see b) and distant growth zones (see d). They must have a particular frequency ***T***_***s***_ of the internal oscillator (see c) which separates them from the other roots (see a-d), including the worst roots (group ***G***_**4**_) which are more likely to have a smaller frequency ***T***_***s***_. Surprisingly, ***G***_**4**_ roots grow towards worse values of ***C*** which suggests that their coordination is efficient, but the sign of **Δ*C*** should be negative. (e, f) Likelihood for a reduced group of 812 roots demonstrating circumnutations (at least one cycle, see S2 Fig), the most successful roots are more likely to have amplitudes of circumnutations in the order of 1D and periods in the order of 1 hour.(TIF)

S1 Video Examples of 2-D and 3-D growth. (MP4)

S2 Video Examples of gravitropic responses. (MP4)

S3 Video Examples of thigmotropic responses. (MP4)

S4 Video Examples of circumnutation movements in 2-D. (MP4)

S5 Video Examples of circumnutation movements in 3-D. (MP4)

S6 Video Examples of root specialization. (MP4)

S1 File (DOCX)
