# Supplementary figures and images for: Conditions for the emergence of circumnutations in plant roots
Source: PLoS One. 2021 May 26;16(5):e0252202. doi: 10.1371/journal.pone.0252202 (PMC8153425; doi:10.1371/journal.pone.0252202)

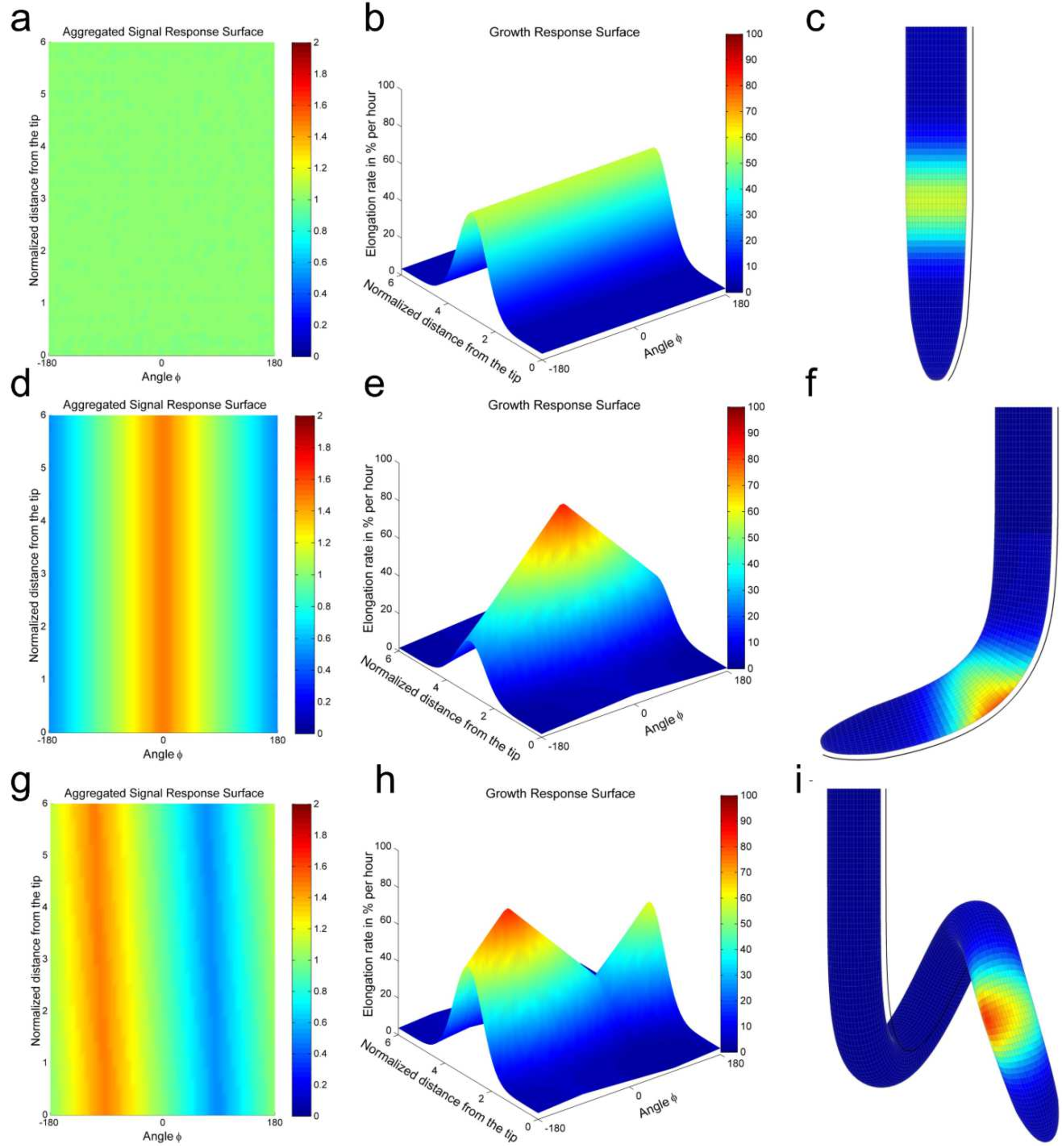

Supplement: S1 Fig — (a) An aggregated signal response surface in time t, distance d from the tip along the center of the root normalized by D, and angle ϕ w.r.t. r, which is used as a reference curve (black lines in (c), (f), and (i)). The present aggregated signal response surface has no inhibited/excited regions. Colors in (a), (d), and (g) show the amplitude of the aggregated signal. (b) A baseline growth response surface defines the zones of faster/slower elongation. Colors in (b), (c), (e), (f), (h), and (i) show the rate (%) of elongation per h. (c) A straight root with the reference curve depicted by a black curve. Aggregated signal response (d) and growth response (e) surfaces after 2 h of excitation signals originating at (d = 0, ϕ = 0) and propagating along the root with a speed of 5D per h lead to a 2-D bending as shown in (f). The same excitation signals originated at d = 0, but with a periodically changing angle ϕ in [−π,+π], as ϕ(t)=π2+tπ10 for t = 0,…,10h (g, h) lead to a 3-D helical growth (i). (TIF) [file pone.0252202.s001.tif]

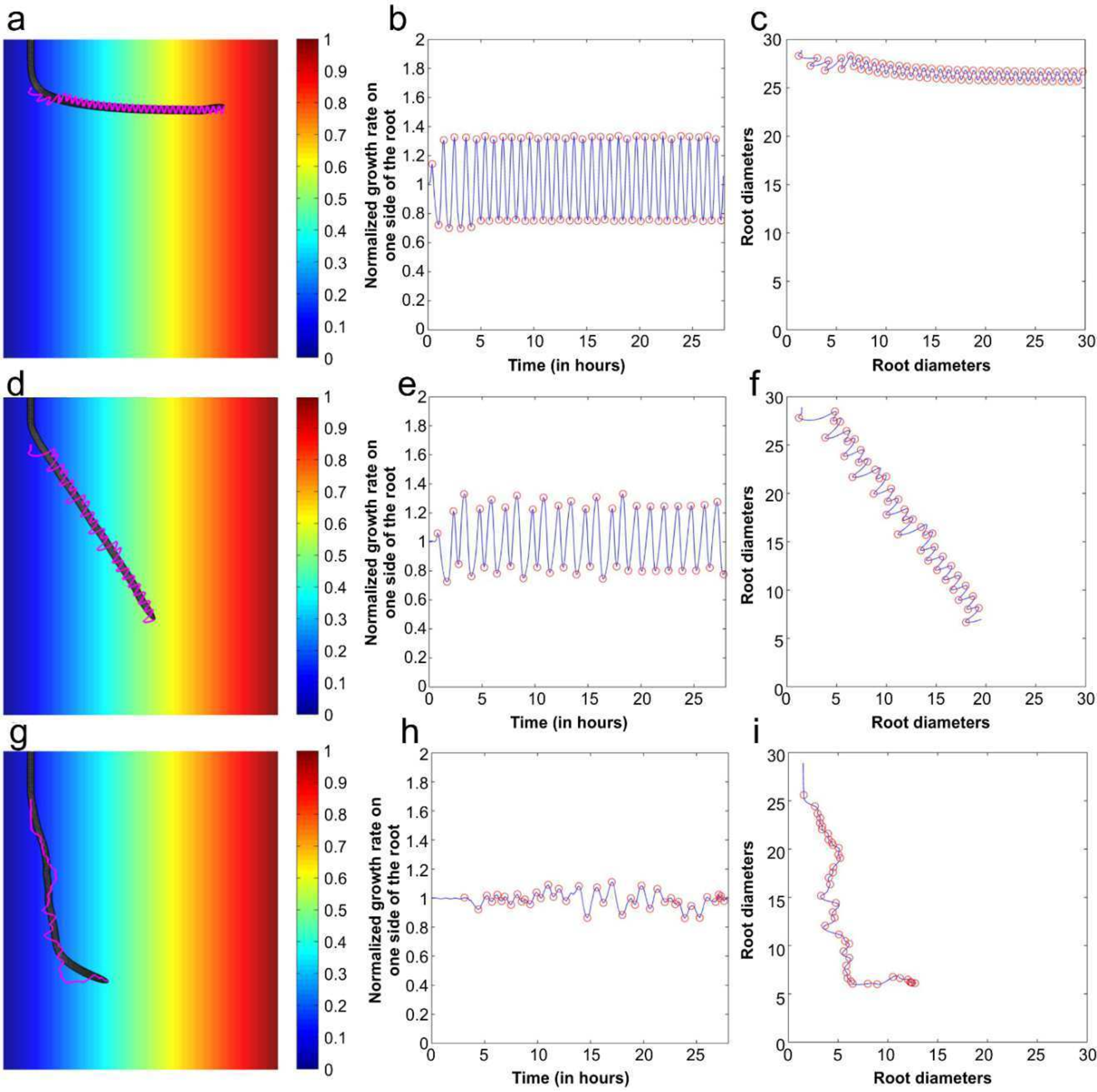

Supplement: S2 Fig — (a, d, g) Three examples of roots performing circumnutation movements driven by an internal oscillatory apparatus. (b, e, h) The normalized growth rate on one side of the root versus time. The red points correspond to the minima and maxima growth rate, and, thus, to the maxima differential growth. The time difference between the red points is used to compute the periods of circumnutation movements. (c, f, i) The red points detected in (b, e, h) are translated into the original coordinate system. By taking any three consecutive red points p1, p2, p3 it is possible to estimate the amplitude of one cycle of circumnutations as ‖p1+p32−p2‖, i.e., the distance between the second point and a mean position between the first and the third points. (TIF) [file pone.0252202.s002.tif]

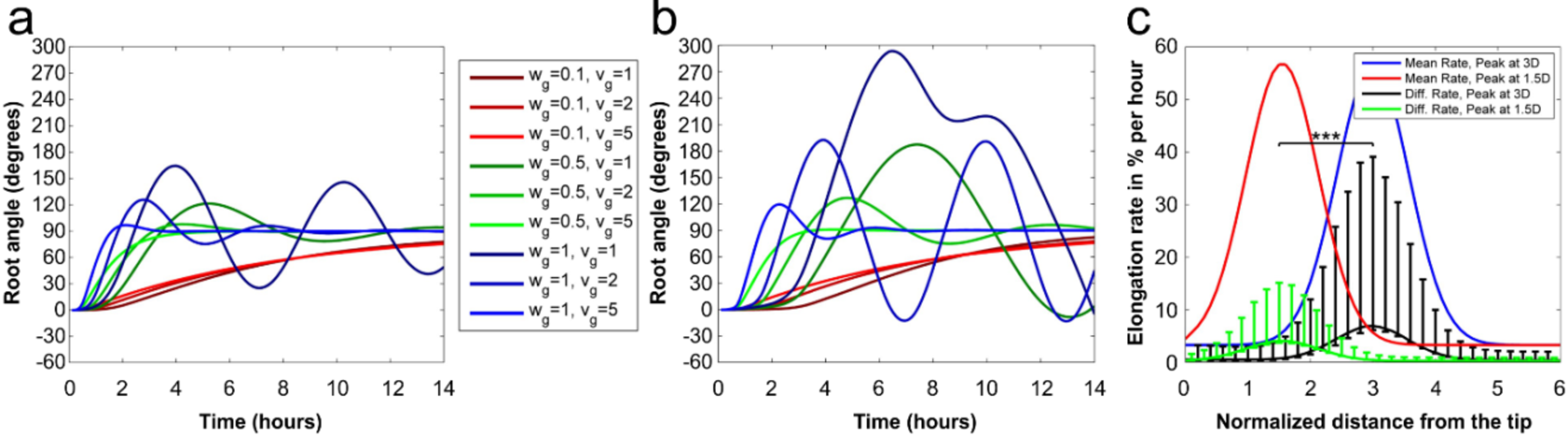

Supplement: S3 Fig — The results in (a) and (b) show the root angle w.r.t. the gravity vector versus time for Fig 4A and 4B, respectively. (c) The mean elongation rates versus distance from the tip for (a) and (b) together with the median, first and third quartiles of differential elongation rates. The twice more distant location of the main growth zones in (b) compared to (a) statistically significant (Wilcoxon rank-sum test, p = 10−9) by a factor of 1.68 increases the median amplitude of differential growth (c), the dynamic change of the latter causes circumnutations. (TIF) [file pone.0252202.s003.tif]

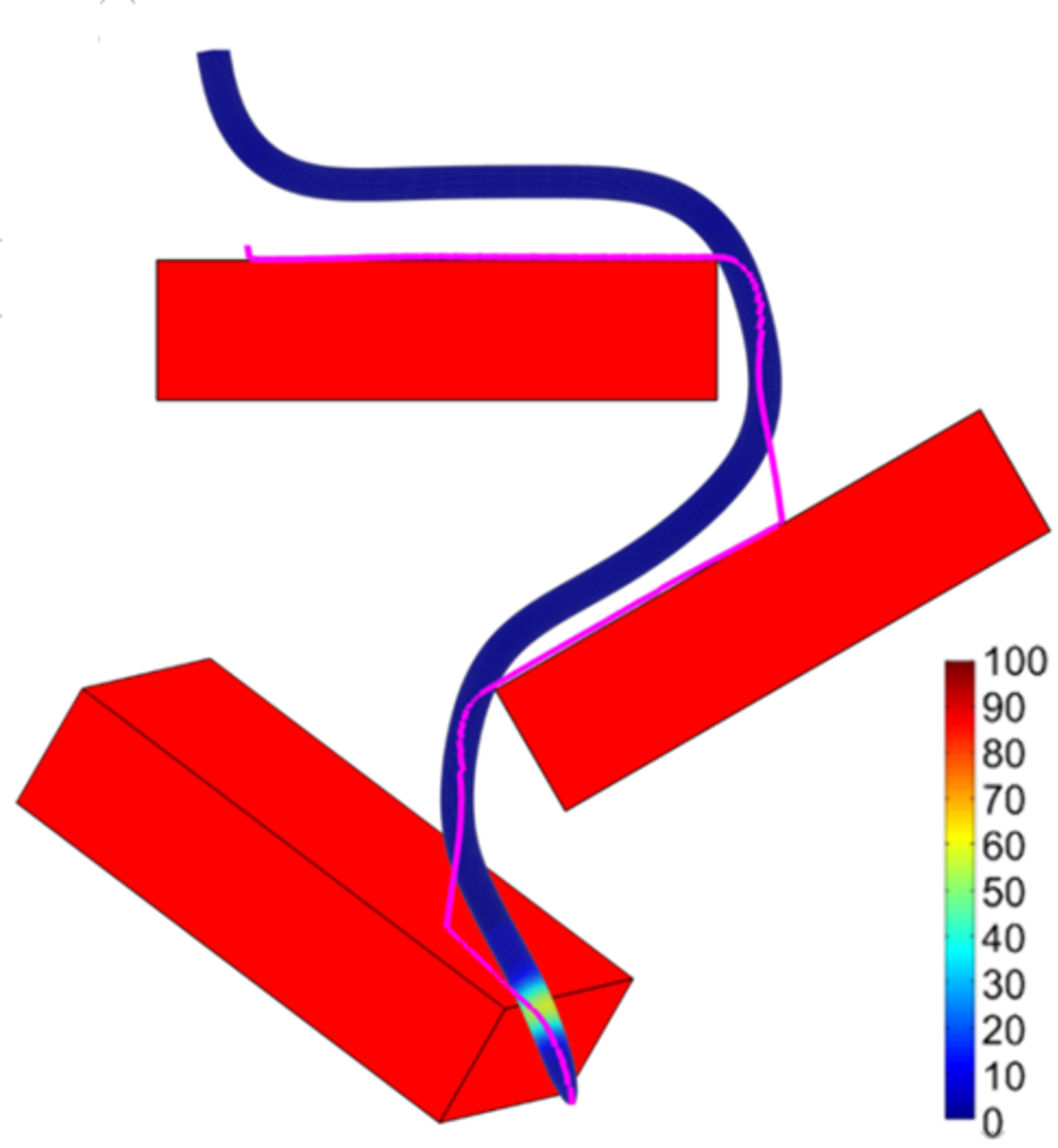

Supplement: S4 Fig — A 3-D example of root interacting with obstacles, with wg = 0.5, wg = 0.5, wt = 0.5. The projections of the root tip trajectories are depicted by magenta lines. (TIF) [file pone.0252202.s004.tif]

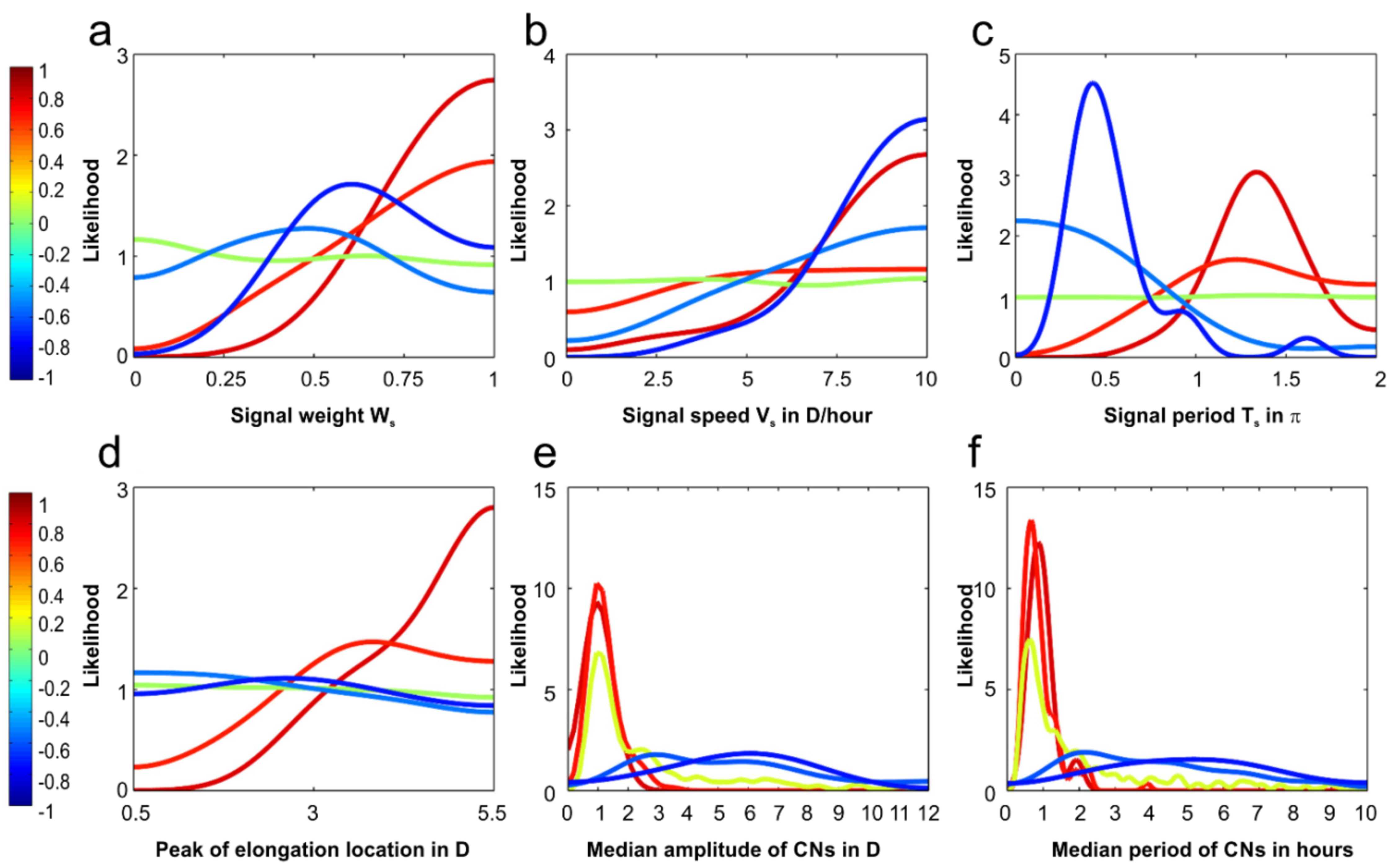

Supplement: S5 Fig — The results of 1,000 experiments with initially vertically oriented roots in a 2-D environment with a linear gradient of C, as shown in Fig 6A–6C. The signaling apparatus of each root was parametrized with uniformly randomly generated stimulus signal weight ws∈[0, 1], speed of signal propagation vs∈[0, 10]D/hour, baseline oscillator frequency Ts∈[0,2π], location of the peak of elongation d∈[0.5, 5.5]D. The performance of each root was measured after 30 simulated hours as f=C(30)−C(0)C*(30)−C(0)∈[−1,1] w.r.t. the best possible C*(30). Ranked after f, 5 non-exclusive groups were selected: G1 with best 5% roots and median f = 0.8156, G2 with best 25% and f = 0.6925, G3 with worst 25% and f = -0.524, G4 with worst 5% and f = -0.706, and G5 with all roots and f = 0.0671. (a-d) The likelihood (measured by kernel density estimation as in [89]) of each group to have specific parameter settings. The best performing roots (group G1, an example is given in Fig 3A) are more likely to have stronger sensitivity to stimulus (see a), faster speed of signal propagation (see b) and distant growth zones (see d). They must have a particular frequency Ts of the internal oscillator (see c) which separates them from the other roots (see a-d), including the worst roots (group G4) which are more likely to have a smaller frequency Ts. Surprisingly, G4 roots grow towards worse values of C which suggests that their coordination is efficient, but the sign of ΔC should be negative. (e, f) Likelihood for a reduced group of 812 roots demonstrating circumnutations (at least one cycle, see S2 Fig), the most successful roots are more likely to have amplitudes of circumnutations in the order of 1D and periods in the order of 1 hour. (TIF) [file pone.0252202.s005.tif]
