# Supplementary material for: Conditions for the emergence of circumnutations in plant roots
Source: PLoS One. 2021 May 26;16(5):e0252202. doi: 10.1371/journal.pone.0252202 (PMC8153425; doi:10.1371/journal.pone.0252202)
Supplement: S1 File — (DOCX) [file pone.0252202.s012.docx]

## Examples of model parametrization

When no excitation/inhibition is present ($S_{i}\left( t,d, \phi\right)=0$), due to the bias $b\left( t,d,\phi\right)=1$and according to eq. (7), the result is $S_{a}\left( t,d,\phi\right)=1$ as shown in S1a Fig.. In this case, for a given $G_{b}\left( t,d,\phi\right)$ and according to eq. (6), the result is $G_{s}\left( t,d,\phi\right)=G_{b}\left( t,d,\phi\right)$ as shown in S1b Fig., which makes the root grow straight as shown S1c Fig..

When signals with $S_{i}\left( t \right)=+1$ at ${\phi=0}$ and $S_{i}\left( t \right)=-1$ at ${\phi=\pm180}$ originate at the tip (${d=0)}$and propagate along the root with speed $v_{1}=\left| v_{1} \right|_{2}$=5D/hour and given an average root growth rate of 1D/hour (i.e., average of $G_{s}\left( t,d,\phi\right)$ over $\left( d,\phi\right)$ is 1D, as roughly the average for the results given in [88]), $S_{a}\left( t,d,\phi\right)$ after ${t=2}$simulated hours corresponds to S1d Fig.. Such signaling pattern leads to a faster growth along ${\phi=0}$ and a bending of the initial root (S1c Fig.) to the root as shown in S1f Fig.. The control of $G_{s}\left( t,d,\phi\right)$ at ${\phi=0}$ and ${\phi=\pm180}$ is sufficient to manipulate the root in 2-D space. Growth control in 3-D space is shown in S1g Fig., where signals with $S_{i}\left( t \right)=+1$ originate at the tip, at a moving ${\phi(t)=\frac{\pi}{2}+\pi t/10}$ for $t=0,\ldots,10$ hours that leads to a 3-D helical growth as shown in S1i Fig..

## Summary of simulations parametrization

**Table 1: Summary of model parameters description and parametrization for the simulations proposed in this study. Parameters that are constant in all simulations are: the time step** $\boldsymbol{\Delta t=0.1}\boldsymbol{hour}$**, the bias** $\boldsymbol{b}\left( \boldsymbol{t,d,\phi} \right)\boldsymbol{=1}$**, the pattern of growth** $\boldsymbol{G}_{\boldsymbol{b}}\boldsymbol{(t,d,\phi)}$ **described by a Gaussian function with a standard deviation equal to 0.1 and peak amplitude of 2D/hour. Only the peak location is varied in simulations. In the table,** $\boldsymbol{\tau}\left( \boldsymbol{t, d, \phi} \right)$ **is the signal decay,** $\boldsymbol{v}_{\boldsymbol{i}}$ **is the signal speed,** $\boldsymbol{w}_{\boldsymbol{i}}\boldsymbol{(t,d,\phi)}$ **is the stimulus weight, where the pedicle** $\boldsymbol{i}$ **can be g for gravity, s for resources, t for touch.**

|  | $\boldsymbol{v}_{\boldsymbol{i}}$ | | $\boldsymbol{w}_{\boldsymbol{i}}\boldsymbol{(t,d,\phi)}$ | | $\boldsymbol{\tau}\left( \boldsymbol{t, d, \phi} \right)$ | | **peak location** | |
| --- | --- | --- | --- | --- | --- | --- | --- | --- |
| **Effects of gravity on circumnutations** | $v_{g}=$  1D/h, 2D/h, 5D/h | | 0.1, 0.5, 1 | | $\Delta t$ | | 1.5D, 3D | |
| **How gravity and mechanical stimuli interact and affect circumnutations** | $v_{g}=v_{t}=$5D/h | | $w_{g}$ = 0.1, 0.3, 0.5, 0.7, 0.9  $w_{t}=1-w_{g}$ | | 0, 10 hours | | 3D | |
| **The role of a possible internal oscillator driving circumnutations** | For this investigation, two new elements are introduced, $T_{s}$, the internal oscillator frequency, and the distance of the oscillatory apparatus (d in the coloum below). Random settings of $v_{s}$, $w_{i}(t,d,\phi)$, $T_{s}$, and d are used. The combinations that are extracted and shown in the main manuscript are: | | | | | | | |
|  | $\boldsymbol{v}_{\boldsymbol{i}}$ | $\boldsymbol{w}_{\boldsymbol{i}}\boldsymbol{(t,d,\phi)}$ | | $\boldsymbol{\tau}\left( \boldsymbol{t, d, \phi} \right)$ | **peak location** | **d** | | $\boldsymbol{T}_{\boldsymbol{s}}$ |
|  | $v_{s}=$5D/h  $v_{g}=0$ | $w_{s}=1$  $w_{g}=0$  $w_{t}=0$ | | $\Delta t$ | 3D | 3D | | $0.75\pi$ |
|  | $v_{s}=$10D/h  $v_{g}=0$ | $w_{s}=0.25$  $w_{g}=0$  $w_{t}=0$ | | $\Delta t$ | 3D | 2D | | $0.94\pi$ |
|  | $v_{s}=$2.5D/h  $v_{g}=0$ | $w_{s}=0.75$  $w_{g}=0$  $w_{t}=0$ | | $\Delta t$ | 3D | 4.5D | | $1.07\pi$ |
|  | $v_{s}= v_{g}=5$D/h | $w_{t}=0$  $w_{s}=0.9$  $w_{g}=0.1$ | | $\Delta t$ | 3D | 0 | | $\pi$ |
|  | $v_{s}= v_{g}=5$D/h | $w_{t}=0$  $w_{s}=0.6$  $w_{g}=0.4$ | | $\Delta t$ | 3D | 0 | | $\pi$ |
|  | $v_{s}= v_{g}=5$D/h | $w_{t}=0$  $w_{s}=0.1$  $w_{g}=0.9$ | | $\Delta t$ | 3D | 0 | | $\pi$ |
|  | $\boldsymbol{v}_{\boldsymbol{i}}$ | | $\boldsymbol{w}_{\boldsymbol{i}}\boldsymbol{(t,d,\phi)}$ | | $\boldsymbol{\tau}\left( \boldsymbol{t, d, \phi} \right)$ | | **peak location** | |
| **How endogenous and exogenous signals act in root specialization** | $v_{s}= v_{g}=v_{t}=5$D/h | | Specialization | | $\Delta t$ | | 3D | |
|  | Specialization:  Crown roots $w_{g}=-1, w_{s}=0.0, w_{t}=0.2$  Primary roots $w_{g}=0.95, w_{s}=0.05, w_{t}=0.2$  Seminal roots $w_{g}=0.05, w_{s}=0.95, w_{t}=0.2$  Lateral roots $w_{g}=0.7, w_{s}=0.3, w_{t}=0.2$ | | | | | | | |
